# Nematode histone H2A variant evolution reveals diverse histories of retention and loss and evidence for conserved core-like variant histone genes

**DOI:** 10.1371/journal.pone.0300190

**Published:** 2024-05-30

**Authors:** Swadha Singh, Noelle Anderson, Diana Chu, Scott W. Roy

**Affiliations:** 1 Quantitative & Systems Biology, University of California, Merced, Merced, California, United States of America; 2 Department of Biology, San Francisco State University, San Francisco, California, United States of America; Laboratoire de Biologie du Développement de Villefranche-sur-Mer, FRANCE

## Abstract

Histone variants are paralogs that replace canonical histones in nucleosomes, often imparting novel functions. However, how histone variants arise and evolve is poorly understood. Reconstruction of histone protein evolution is challenging due to large differences in evolutionary rates across gene lineages and sites. Here we used intron position data from 108 nematode genomes in combination with amino acid sequence data to find disparate evolutionary histories of the three H2A variants found in *Caenorhabditis elegans*: the ancient H2A.Z^HTZ-1^, the sperm-specific HTAS-1, and HIS-35, which differs from the canonical S-phase H2A by a single glycine-to-alanine C-terminal change. Although the H2A.Z^HTZ-1^ protein sequence is highly conserved, its gene exhibits recurrent intron gain and loss. This pattern suggests that specific intron sequences or positions may not be important to H2A.Z functionality. For HTAS-1 and HIS-35, we find variant-specific intron positions that are conserved across species. Patterns of intron position conservation indicate that the sperm-specific variant HTAS-1 arose more recently in the ancestor of a subset of *Caenorhabditis* species, while HIS-35 arose in the ancestor of *Caenorhabditis* and its sister group, including the genus *Diploscapter*. HIS-35 exhibits gene retention in some descendent lineages but gene loss in others, suggesting that histone variant use or functionality can be highly flexible. Surprisingly, we find the single amino acid differentiating HIS-35 from core H2A is ancestral and common across canonical *Caenorhabditis* H2A sequences. Thus, we speculate that the role of HIS-35 lies not in encoding a functionally distinct protein, but instead in enabling H2A expression across the cell cycle or in distinct tissues. This work illustrates how genes encoding such partially-redundant functions may be advantageous yet relatively replaceable over evolutionary timescales, consistent with the patchwork pattern of retention and loss of both genes. Our study shows the utility of intron positions for reconstructing evolutionary histories of gene families, particularly those undergoing idiosyncratic sequence evolution.

## Introduction

All characterized eukaryotic cells compact their DNA into nucleosomes, which consist of an octameric histone complex comprised of two copies each of the core histone proteins H2A, H2B, H3, and H4, and which wrap around ~147 bps of DNA [1–4]. In many species, genes encoding canonical core histones (also referred to as ‘replication-dependent histones’) do not contain introns and are organized into multiple gene copies found in tandem-repeat arrays, which facilitates their rapid, coordinated expression [4–7]. The expression of canonical histones is tightly coupled with the S-phase of the cell cycle because of the critical need for the large bulk of histone proteins required to package and compact the newly synthesized DNA [8–10].

Histones have evolved variant forms that further regulate chromatin compaction and affect processes including transcription, DNA repair, and development [11–20]. Such histone variants are often considered to be a part of the ‘histone code’ and control distinct sets of genes during specific times and tissues in both animals and plants [11, 12, 21–23]. Among all the core histones, H2A is the most evolutionarily dynamic: (i) core H2A shows more sequence turnover over evolutionary time; and (ii) H2A shows a larger number of variant gene copies across eukaryotes, which often show variation in expression patterns [24, 25]. For example, the highly conserved H2A.Z is present across eukaryotic diversity and is ubiquitously expressed. It has been shown in different species to play a variety of roles including transcriptional regulation, heterochromatin boundaries, DNA repair, DNA replication, and dosage compensation [26–31]. In contrast, H2A.X has arisen independently across several eukaryotic lineages, with functions in DNA damage response and transcription [4, 18, 32], while macroH2A, which is involved in X inactivation and stress response, is present in vertebrates and some invertebrate lineages [3, 24, 33–36]. Finally, short histone variants, such as H2A.B, H2A.P, H2A.L, and H2A.Q, are expressed in various eutherian mammalian testis (H2A.B is also expressed in brain cells) [25, 37–39]. In short, a great wealth of histone H2A variants are observed, ranging from those shared across all eukaryotes to others that are species-specific [3, 4, 18, 19, 35]. H2A variants also have distinct functions in a broad range of processes and can be ubiquitously expressed or expressed only in certain tissues [11, 25, 40, 41]. With the exception of a few well-studied variants like H2A.Z, how histone H2A variants have arisen and evolved remains understudied.

Clues to how histone variants differ may stem from the distinct gene structures and expression patterns, which contrast with canonical histones. Unlike canonical histone genes, variant histone gene expression is not restricted to S phase expression and can be tissue-specific. Most histone variants are typically found in a single copy in the genome and contain introns in their pre-mRNAs [5–7, 42, 43]. Notably, the roles of these introns in variant function remain poorly studied, including whether the introns themselves impart novel functionality. For instance, sequences found within introns of other genes can regulate gene expression [42, 44–47]. Introns can also allow for alternative splicing, which is common in animals and plays an important role in development and disease [48–51]. Introns have been used in other studies to determine the evolutionary trajectory of other protein families [52–56]. Thus, the presence of introns in histone variants may provide useful clues to exploring the histone evolution and function across closely related species.

Core histone functions are expected to be highly conserved across eukaryotes, given their central roles in ensuring DNA packaging and protection [4, 57]. Thus, observed protein changes that have occurred through evolution in core histones are expected to be largely neutral with respect to protein function. On the other hand, amino acid differences between variant histones and core histones are thought to generally lead to functional differences [3, 4, 58]. Indeed, protein sequence differences between well-studied variants and their core homologs have been shown to affect chromatin structure and function. The histone H3.3 variants of animals, for example, which differ from each other by 4–5 amino acids, play distinct roles in transcriptional activation, chromatin remodeling, heterochromatin formation, and development [14, 16, 59–62]. On the other hand, the functional significance of protein differences between variant histones and their core counterparts in some cases is unclear. For instance, the protein sequence of *C*. *elegans* HIS-35 differs by a single ‘A’ instead of a ‘G’ at the 124th position from the S-phase *C*. *elegans* H2A, the functional significance of which is unknown.

**Fig 1:**
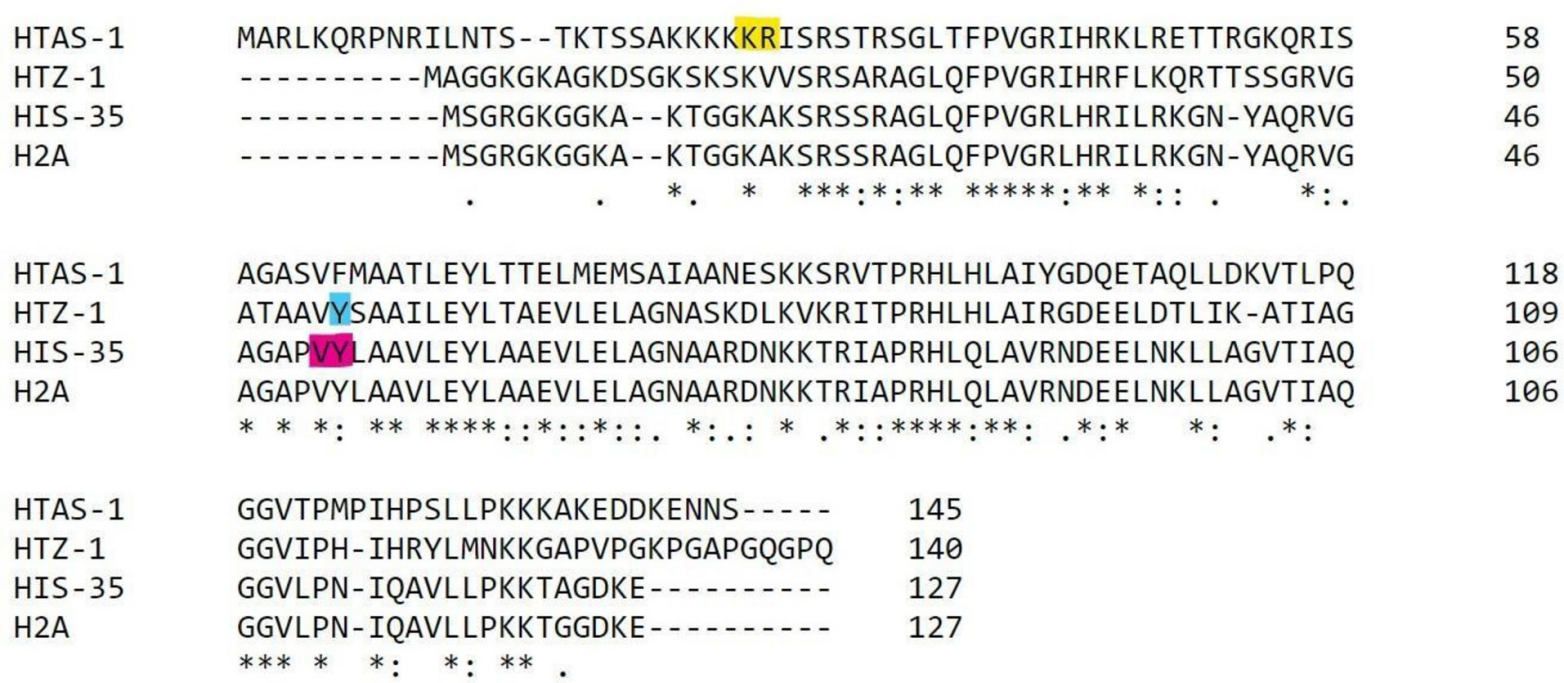
Sequence and intron position comparison of three H2A variants of *Caenorhabditis elegans*. C. elegans core histone H2A and its three variant paralogs contain different sequences and intron positions. The variants differ either in intron positions or the phases in which they interrupt the codon. HTAS-1 (highlighted with a yellow) has a phase 0 intron between the 26th and 27th amino acid; HTZ-1 (highlighted with blue) has a phase 2 intron splitting the 57th amino acid; HIS-35 (highlighted with pink) has a phase 0 intron between 50th and 51st amino acid.

In this study, we focus on the evolutionary histories of the three very different H2A variants found in *Caenorhabditis elegans*: H2A.Z^HTZ-1^, HIS-35, and HTAS-1 (Fig 1). H2A.Z^HTZ-1^ is an ortholog of the evolutionarily conserved variant, H2A.Z. HIS-35 is poorly characterized and intriguingly differs from core H2A by a single amino acid difference. HTAS-1 shows a greater divergence from core H2A, particularly at the highly divergent C and N termini, appears to be expressed only in sperm, and has only been reported in *C*. *elegans* to date [63]. The presence of a discrete number of H2A variants with distinct functions and/or expression patterns, in combination with the availability of sequence data across a large number of nematode species, allows a unique opportunity to track the evolutionary trajectory of this histone variant family.

## Results

### H2A family evolution poses challenges to standard phylogenetic methods

We sought to understand the evolutionary history of the three H2A variants found in the *C*. *elegans* genome. Taking advantage of vast sequencing [64–66] efforts across nematode species, we used BLAST searches across 108 reported nematode genomes to identify all annotated copies of H2A and H2A-related gene variants across the diversity of nematodes. After filtering and collapsing identical proteins, we were left with 593 nucleotide sequences and 408 unique protein sequences. Interestingly, these sequences contain additional intron-containing copies absent from *Caenorhabditis* nematodes, some of which likely represent additional *de novo* evolution of H2A variants in other nematode lineages. However, preliminary scrutiny of these genes revealed high rates of likely annotation errors (for instance, chimeric gene copies generated by incorrect intron calls). Therefore, we opted to focus on the three confidently characterized nematode H2A variants, namely the three found in *C*. *elegans*.

We then used Maximum Likelihood phylogenetic methods to reconstruct the evolutionary history of these sequences (S2 Fig in S2 File protein-based and S3 Fig in S2 File nucleotide-based; intronless sequences are candidate core/canonical histones). However, scrutiny of the recovered phylogenetic trees revealed several aberrant findings. For instance, putative intronless core H2A proteins were grouped as separate clades that included very deeply-diverged nematode sequences; on the other hand, species-specific variants were often found grouped far from core proteins from the same or related species, despite that they are expected to have evolved recently from the core genes. Perhaps the clearest case arose from performing phylogenetic reconstruction on core H2A orthologs along with the HIS-35 orthologs identified based on intron positions (described below). Here, we expect distinct clades of H2A and HIS-35 sequences, yet we recover no such separate clades (S4 Fig in S2 File, HIS-35 in yellow).

Some of these anomalies are as expected by errors in phylogenetic reconstruction due to model misspecification (the phenomenon in which differences between the assumed model of sequence evolution and the actual evolutionary process lead to errors in phylogenetic reconstruction) [67]. The possibility of model misspecification is elevated in the case of histone genes by several factors: extreme differences in rates across sites (some sites show conservation across eukaryotes, others are variable within the genus of *Caenorhabditis*); large relative differences in rates across gene lineages (the extremely slow evolution of core proteins, but substantially higher rates of change in some variants); and the generally small number of total sites, given the short length of histone proteins (which makes estimation of accurate sequence evolution models challenging). Notably, it could also be possible that grouping of paralogs truly reflects sequence evolution–in particular, if there is recombination between core and variant histones (i.e., gene conversion), core and variant histone sequences could be expected to group together. However, the result for our purposes of identifying variant orthologs is the same, namely a failure to clearly distinguish variant and core histones.

### Intron position and phase help to distinguish the three H2A variants of *C*. *elegans*

As an alternative approach, we used another potential source of phylogenetic information: the positions at which the spliceosomal introns interrupt nuclear gene copies. Introns are largely absent from core histones, but are often present in variant histones. Intron positions can be conserved over very long evolutionary time periods in orthologous genes [52–55, 68, 69]. Whereas early work considered the possibility of intron ‘sliding’, in which an intron would migrate a few base pairs along a gene, recent work has shown that intron sliding is a very rare occurrence, and that intron positions are very often conserved over very long times [53, 69]. For example, in *Theileria* apicomplexans, 99.7% of intron positions are conserved between *T*. *parva* and *T*. *annulata*, diverging roughly 82 million years ago [52]. Similarly, in mammals, 99.9% of intron positions are conserved between humans and dogs, diverging around 100 million years ago [54].

We began our study of intron-exon structures in H2A variants by obtaining intron-exon structures for all H2A gene family members and performing alignments to determine intron positions shared across genes. We first aligned the three main *C*. *elegans* H2A variants (Fig 1), HIS-35, HTAS-1, and H2A.Z^HTZ-1^, each of which has a single intron position (highlighted boxes in Fig 1). Scrutiny of the intron positions showed that the three genes contain introns at different positions, differing in the codon that they interrupt or and/or in the phase at which they disrupt the codon. Interestingly, the intron position in HIS-35 falls very near to that found in H2A.Z^HTZ-1^. However, these introns are unlikely to represent a shared intron, since they are found in different phases, and substantial work indicates that intron positions rarely slide between phases [53, 69]. Moreover, it is unlikely that HIS-35 arose from H2A.Z rather than from H2A given the near identity of *C*. *elegans* HIS-35 to core H2A and only 60% sequence identity to H2A.Z^HTZ-1^: for HIS-35 to have evolved from H2A.Z^HTZ-1^ would require 47 precise amino acid identity changes from the H2A.Z^HTZ-1^ sequence to the H2A-like sequence HIS-35. We wondered whether the intron insertion sites show similarities to the so-called “protosplice site” (AG|GT), as predicted by a variety of intron gain mechanisms. We found that introns in both HIS-35 and HTAS-1 do show signal, aligning to CA|GT and AG|GC sites, respectively, in *C*. *elegans* H2A. The HTZ-1 intron shows no such correspondence CT|GG, perhaps reflecting its greater evolutionary age (see below).

### Intron position and sequence evidence indicates the origin of HTAS-1 within *Caenorhabditis* and subsequent gene retention and loss

**Fig 2:**
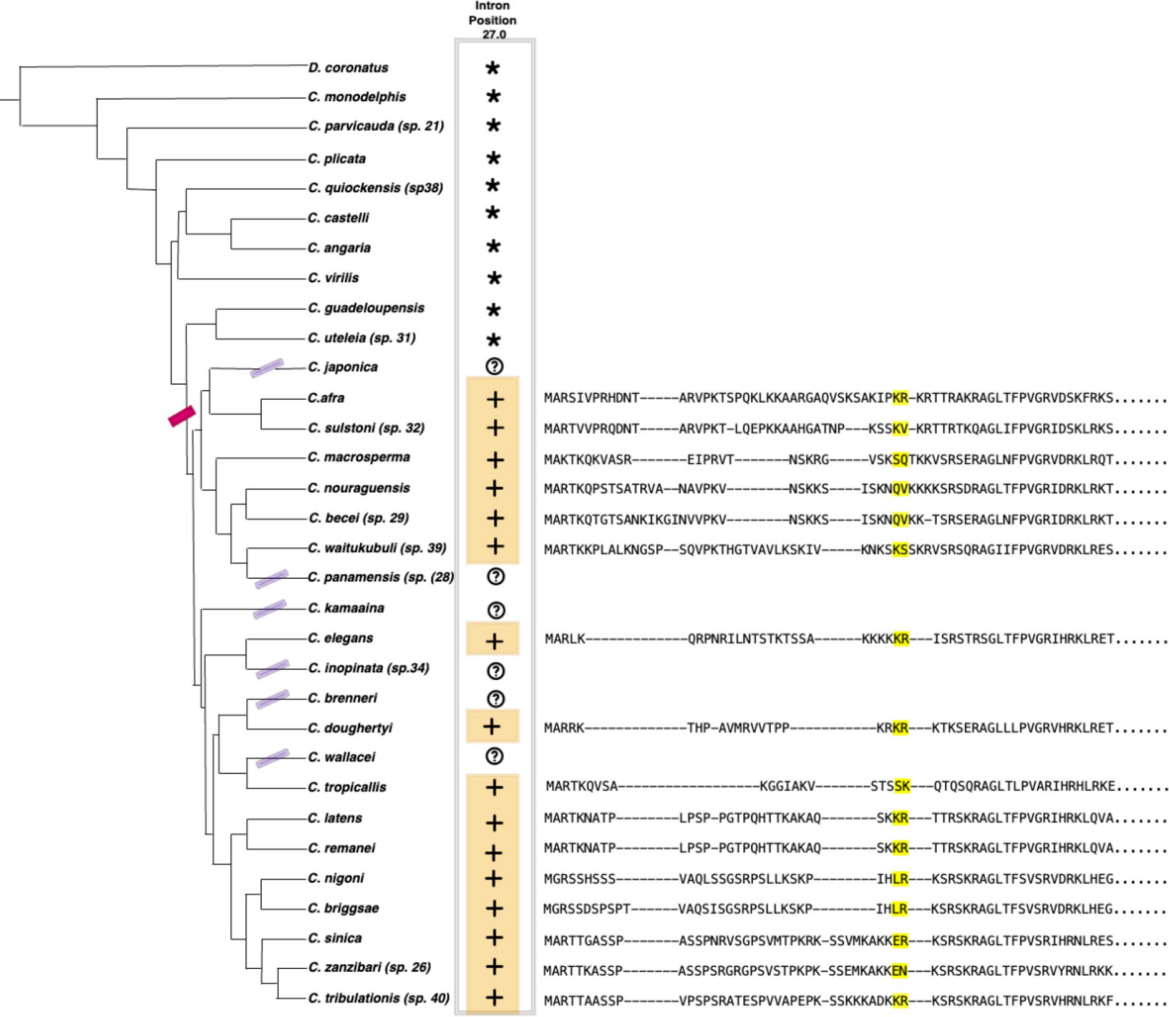
Intron position and sequence evidence indicate the origin of HTAS-1 within *Caenorhabditis* and subsequent loss. On the left is the previously reconstructed species tree topology for Caenorhabditis species and outgroup Diploscapter coronatus [65]. The likely origin of HTAS-1 is indicated with a dark pink bar. HTAS-1 presence in a species is indicated by the plus sign. The question mark denotes species where HTAS-1 homologs could not be identified. The species where HTAS-1 was not incorporated are indicated as an asterisk. On the right is the multiple sequence alignment of HTAS-1 proteins showing the aligned intron positions (highlighted with yellow).

The *C*. *elegans* sperm-specific H2A variant, HTAS-1, contains a single intron between codons 26 and 27 (“phase 0”; Fig 1, highlighted with yellow). Alignment across all the H2A variants of 108 nematodes revealed 16 genes that share an intron at the exact homologous position as *C*. *elegans* HTAS-1 (Fig 2), all from species falling within a single subclade of 22 species (represented by the red hash mark on the tree branch in Fig 2) within the *Caenorhabditis* genus, suggesting an origin of this intron position within the common ancestor of this clade. Scrutiny of the sequence gene tree (S2 and S3 Figs in S2 File, highlighted in green, IP_152, and green circles) revealed that this same set of genes (i.e., those sharing the intron) appears together as a clade, reinforcing the notion that the genes sharing the HTAS-1 intron position represent a set of orthologs. We next sought to identify potential HTAS-1 orthologs in additional *Caenorhabditis* species, both the 6/22 within the HTAS-1-containing subclade that lack intron-containing HTAS-1 candidates (marked by purple hash on the tree branch in Fig 2) as well as the 10 species outside this subclade. No genes from any such species were found grouping with the putative HTAS-1 ortholog clade; thus, overall, we could not find any other genes that are candidates for HTAS-1 orthologs. In total, the data is consistent with the origin of HTAS-1 and its gene-specific intron occurring in the same ancestor of a subset of *Caenorhabditis* species.

The 6/22 species in the clade that lack clear HTAS-1 orthologs could represent either gene loss or annotation failure (particularly plausible for short genes such as histones) or some combination thereof. We performed TBLASTN searches against the 6 genomes to identify missed HTAS-1 orthologs but did not find additional candidates. However, such searches may also have low sensitivity.

Because of the much higher rate of evolution of HTAS-1, HTAS-1 from one species often shows a greater degree of similarity to the core H2A sequence from another species than to its HTAS-1 ortholog (e.g., HTAS from non-*elegans* species has similar % identity (% identity within 5%) to H2A as to HTAS for 9/16 species.). Consequently, TBLASTN searches using HTAS-1 from one species typically give many hits, with the likely HTAS-1 not being within the top dozen hits, making it difficult to find unannotated HTAS-1 candidates or to confidently infer their absence. In total, then, the data is consistent with a single origin of HTAS-1 and its characteristic intron position within the ancestor of a subset of studied *Caenorhabditis* species, with potential losses in up to six independent lineages (Fig 2).

### Intron position conservation suggests HIS-35 originated in the *Caenorhabditis-Diploscapter* ancestor and has experienced subsequent gene retention and loss

**Fig 3:**
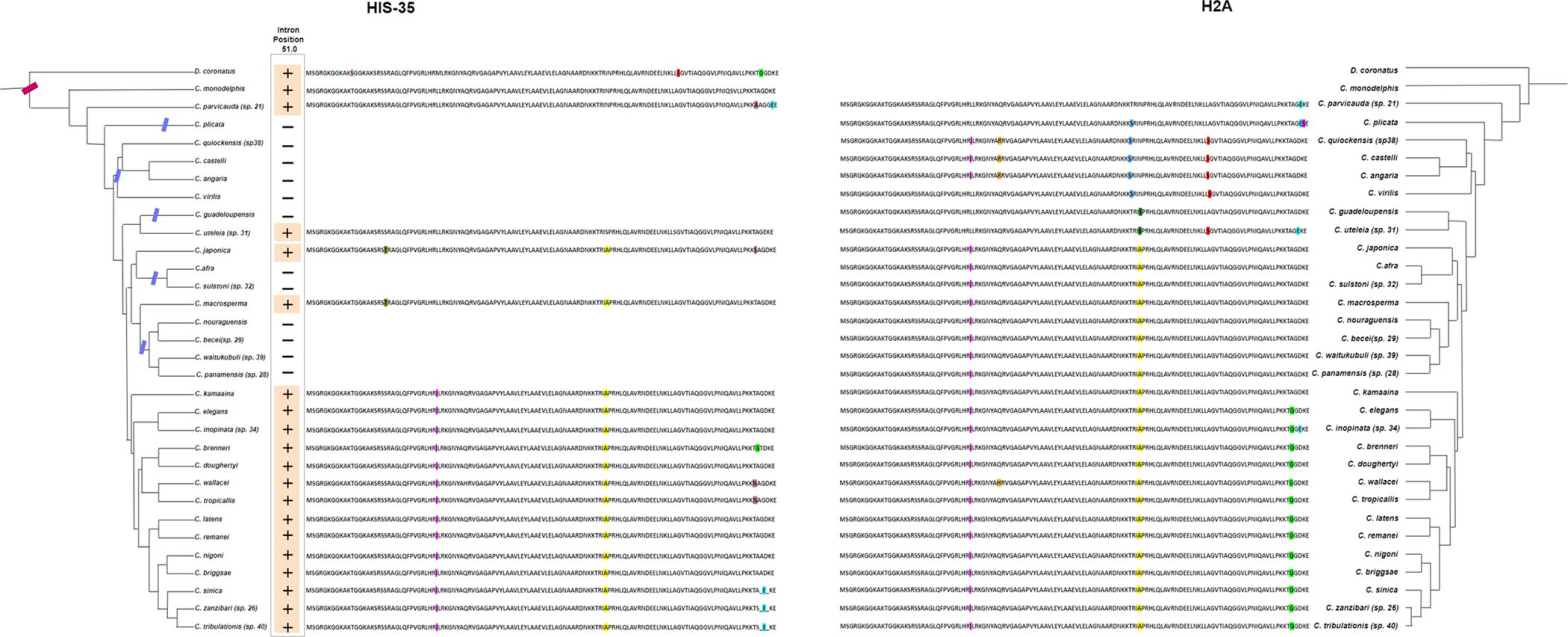
Comparison of the variant HIS-35 (left) with canonical H2A (right) sequences across Caenorhabditis species and the Diploscapter coronatus outgroup. Species tree cladograms for Caenorhabditis species and Diploscapter coronatus are based on reference 68. Inferred sequence changes relative to the reconstructed ancestral sequence are highlighted with different colors, with identical changes colored the same across the two trees (i.e., the L-to-I change at position 35 is colored fuchsia on both protein alignments).

The variant HIS-35 of *C*. *elegans* has a phase zero intron between the 50th and the 51st codon (Fig 1). Alignment across all H2A variants revealed 20 species with genes that share this intron position (Fig 3, marked with a plus sign). In all cases, these genes show very high sequence similarity with H2A at the protein level. These genes are from species falling in the clades of *Caenorhabditis* and its sister genus *Diploscapter*, representing 20/32 species within this clade, suggesting an origin of this intron position within the common ancestor of *Diploscapter* and *Caenorhabditis* (Fig 3).

We sought to determine whether the 12 descendent species without a clear candidate HIS-35 gene represent true losses or merely failures of gene annotation. TBLASTN and BLASTN searches failed to recover H2A copies containing the HIS-35-characteristic intron position. In contrast to the case of HTAS-1 above, we are confident in the ability of these methods to detect HIS-35 orthologs, since we were able to detect the annotated HIS-35 ortholog by identical methods (i.e., the positive control). For instance, using *C*. *japonica* HIS-35 as a query, BLASTN searches revealed the (annotated) putative ortholog in *C*. *macrosperma*, but did not reveal unannotated candidate orthologs in *C*. *nouroguensis*, *C*. *waitukubuli (sp*. *39)*, *C*. *panamensis (sp*. *28)*, or *C*. *becei (sp*. *29)*, which are equally closely related to *C*. *japonica*. In addition, whereas species lacking HTAS-1 appear to be randomly scattered across the HTAS-1-containing clade, as expected by random gene annotation failures, species lacking HIS-35 within the HIS-35-containing clade group in subclades. This is as expected by true biological loss and not by random annotation failures. We additionally used BUSCO v5.5.0 [70] on *Caenorhabditis species* and the outgroup *Diploscaptor coronatus* to assess genome assembly quality and completeness using the nematode odb10 database for comparison. To test the possibility that inferred gene losses of HIS-35 and HTAS-1 in various *Caenorhabditis* species are the result of poor genome assemblies rather than legitimate loss, we conducted one-tailed t-tests on both BUSCO completeness and missing gene estimates. When testing if there is either fewer complete genes or more missing in the assemblies of those with inferred gene losses compared to those without (from Figs 2 and 3-left), we do not find that species with inferred losses have poorer quality genome annotations (S1 Fig in S2 File) (HTAS-1: Completeness (p = 0.3243), Missing (p = 0.3605); HIS-35: Completeness (p = 0.4203), Missing (p = 0.4781)). Thus, we conclude that HIS-35 has been lost some 5 times independently in different *Caenorhabditis* lineages.

### Conservative protein evolution of HIS-35 and evidence for occasional gene conversion with core H2A

HIS-35 provides a particularly interesting example for histone protein evolution. In *C*. *elegans*, the protein sequence of HIS-35 differs by just one amino acid from the S-phase H2A despite the substantial evolutionary time since divergence. Namely, HIS-35 has an “A”, while H2A has a “G”, at position 124 of the amino acid sequence. It is possible that even the single amino acid change G->A may alter the function of the HIS-35 variant relative to H2A.

However, when we looked at position 124 in the canonical H2A sequences of all the *Caenorhabditis* species, we actually found that HIS-35-like “A” in many species, in a phylogenetic pattern suggesting that out that the “A” is ancestral to canonical H2A (Fig 3). The presence of “A” at position 124 in extant and ancestral *Caenorhabditis* H2A suggests that HIS-35 likely has not diverged in function from the ancestral H2A. Also consistent with a lack of general differentiation in protein function between H2A and HIS-35, we also found that the encoded protein sequences of HIS-35 and H2A of species *C*. *kamaaina* are exactly the same (Fig 3). These findings are not as expected if canonical H2A and HIS-35 proteins have different functions, but are as expected if the proteins are functionally equivalent.

The multiple copies of core histone genes are known to undergo so-called concerted evolution, with sequences being transferred between gene copies by gene conversion [71–74]. We wondered whether concerted evolution plays a role in the identical protein sequence changes observed in the H2A and HIS-35 paralogs of some species. Under concerted evolution, the two sequences undergoing concerted evolution are homogenized (one overwrites the other). Consequently, the prediction is that such events should lead the interconverting partners to the group together on a phylogenetic tree. To search for evidence of concerted evolution, we reconstructed separate phylogenetic trees of exon 1 and exon 2 for all H2A and HIS-35 *Caenorhabditis* sequences. Most of the reconstructed tree largely reflected the species tree, suggesting against the possibility of widespread concerted evolution (S5 and S6 Figs in S2 File). However, we did observe the grouping of the two gene sequences for *Caenorhabditis parvicauda* (sp. 21), consistent with the concerted evolution of HIS-35 and H2A in this species. Concerted evolution of these genes is consistent with a lack of functional differentiation of the encoded proteins, though admittedly the low rate of such events weakens the strength of this argument.

### The intron loss and gain in HTZ-1 show a dynamic evolutionary history

**Fig 4:**
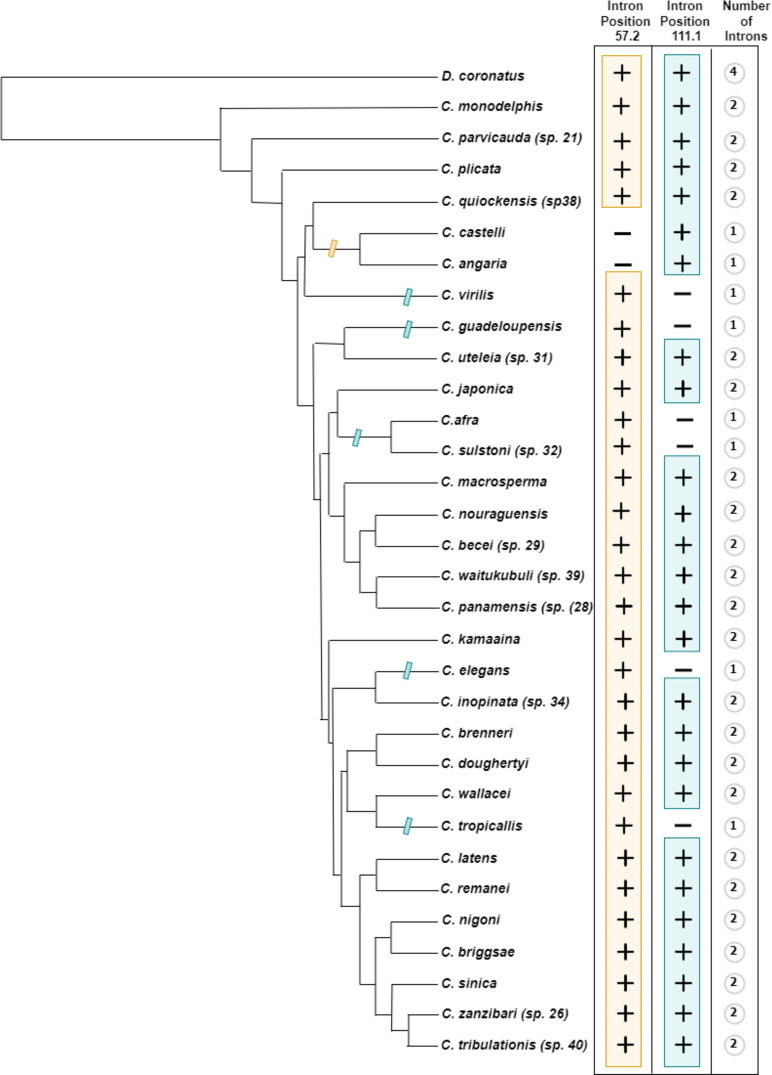
The dynamic history of intron loss and gain in HTZ-1. On the left is the previously reconstructed species tree topology for Caenorhabditis species and Diploscapter coronatus [65]. HTZ-1 characteristic intron presence and absence in a species is indicated by the plus and minus signs. The yellow hash mark on the tree branch depict the loss of intron-1 whereas the blue hash marks on the branch suggests the loss of intron 2 in those lineages.

The ubiquitously expressed *C*. *elegans* H2A variant H2A.Z^HTZ-1^ is the ortholog of H2A.Z, which is evolutionary conserved across eukaryotes [3, 75]. *C*. *elegans* H2A.Z^HTZ-1^ has an intron that splits the 57th codon at position 2 (Fig 1). Within the alignment across all H2A variants, we searched for genes that share the single intron position of *C*. *elegans* H2A.Z^HTZ-1^, revealing 30 genes that share this position (Fig 4, marked with a plus sign; S2 and S3 Figs in S2 File). These 30 genes are putative H2A.Z^HTZ-1^ orthologs. Consistent with their orthology, these 30 genes grouped together on the tree. Unexpectedly, we found that 22 of these 30 putative H2A.Z^HTZ-1^ genes have two (or more in *Diploscapter*) introns in their genes, one at position 57 in phase 2 and the other at position 111 in phase 1. Both introns have been repeatedly individually lost in different lineages (including in the lineage leading to the single-intron *C*. *elegans* H2A.Z^HTZ-1^ gene). Species including *C*. *elegans*, *C*. *tropicalis*, *C*. *sulstoni (sp*. *32)*, *C*. *afra*, *C*. *guadalupensis*, *C*. *virilis* have lost the second H2A.Z intron which is at position 111.1, whereas *C*. *castelli* and *C*. *angaria* have lost their first intron. These results are consistent with general results in protein-coding genes, wherein intron loss is common across the *Caenorhabditis* phylogeny [75, 76]. Nonetheless, the finding that general trends of intron loss may equally apply to histone variant genes is important in understanding the functional implications of variant introns.

Intron presence is a conspicuous difference between core and variant histones, raising the question of the functional significance of variant histone introns: specifically, are variant histone introns important for differentiating the specific functions of those variants from their core paralogs? The finding of recurrent loss of introns from a variant gene suggests that the specific positions or sequences of introns in a particular histone variant gene may not be of particular functional importance. Nonetheless, it is of note that all observed H2A.Z orthologs contain at least one intron, suggesting that the presence of at least one intron, at whatever position, could be important, for instance for efficient expression of variant histone genes, consistent with their expression through canonical gene expression pathways, in which intron presence often promotes gene expression.

## Discussion

### Introns as sources of phylogenetic information

This study is among the first to leverage the information present in intron positions to decipher the evolutionary history of histone variants [77]. Previous studies have shown intron position conservation among widely diverged eukaryotic species [52–54, 56, 68, 69, 77]. For instance, intron positions are highly conserved between humans, mice, and fish [56]. Thus, intron positions contain a record of evolutionary history that can facilitate insights into gene history. The utility of introns here differs across variants and allows separate questions to be addressed. The clearest case comes from HIS-35, in which nearly complete evolutionary conservation of H2A and HIS-35 sequences leads to a lack of phylogenetic signal within proteins. At the other end of the spectrum lies HTAS-1, which shows much more rapid sequence evolution. However, here, the discrepancy in rates between HTAS-1 and H2A, and likely between different sites in the two proteins (i.e., the N and C termini are highly constrained in H2A but fast-evolving for HTAS-1) makes it impossible to define a single evolutionary model across the gene family. As expected by general long branch attraction considerations, this leads to fast-evolving HTAS-1 incorrectly grouping far away from *Caenorhabditis* core H2A sequences. This generally undermines our confidence in sequence-based phylogenetic reconstruction of HTAS-1 genes. Interestingly, intron-defined putative HTAS-1 orthologs do group as a clade, indicating that sequence-based phylogenetic reconstruction was likely successful for this group; however, without the intron position information, our general lack of confidence in the methods’ success for HTAS-1 would lead us to question this finding. Thus, for this case, even though the sequence-based phylogenetic methods apparently correctly identified the HTAS-1 clade, the orthologous information from intron positions was necessary for us to be confident in the sequence-based phylogeny. Intron positions were also indispensable in distinguishing the origins of HTAS-1. Because HTAS-1 arose in an ancestor containing two genes encoding nearly identical proteins (H2A and HIS-35), it is difficult to determine whether H2A evolved from the preexisting variant HIS-35 or *de novo* from H2A. The fact that all candidate HTAS-1 genes lack the HIS-35 intron suggests *de novo* evolution from H2A, not HIS-35 (though intron loss cannot be excluded).

### Evidence from phylogenetic analysis for functional significance of recently-evolved histone variants

In addition to tracing the origins and subsequent history of gene loss and retention, our results provide insights into the possible functions of histone H2A variants. For example, HIS-35 differs by a single amino acid from the S-phase H2A (‘A’ instead of ‘G’ at the 124th position). Given that characterized histone variants are thought to largely represent functionally distinct proteins, one hypothesis is that this single difference functionally differentiates HIS-35 protein from H2A. One way that a single amino acid change could have outsize effects is through altering the landscape of posttranslational modifications, which are key to histone function. For instance, this is the case with the H3 variants H3.1 and H3.2 which differ from one another by single amino acid and show distinct patterns of expression and post-translational modifications [60, 61].

However, examination of position 124 across H2A sequences of all the *Caenorhabditis* species revealed that the ‘A’ at position 124 found in HIS-35 is ancestral. Considering the presence of an A at position 124 in other canonical H2As suggests the variant HIS-35 might have the same function as the canonical H2A. Lack of functional differentiation is also consistent with the similarity of A and G amino acids. This hypothesis is also supported by the case of *C*. *kamaaina*, in which the encoded HIS-35 and H2A protein sequences are exactly the same. While these results do not disprove the hypothesis that H2A and HIS-35 encode proteins with important functional differences (except in the case of *C*. *kamaaina*), we propose instead that the functional importance of HIS-35 protein lies in allowing for the expression of a protein with overlapping or redundant functions to canonical H2A that is not restricted to S-phase, as is the case with canonical H2A. This could allow expression in differentiated cells that do not undergo mitosis and enable tissue-specific expression. Such a potential semi-redundancy could help to explain the ambivalent phylogenetic pattern, in which retention of HIS-35 in most species suggests functional importance whereas loss in 5 independent lineages suggests conditional expendability. Interestingly, a similar pattern of lineage-specific loss has been observed for H2B variants, which in that case has been interpreted as encoding functionally important but partially redundant functions [78].

### Function in reproduction for the sperm-specific variant HTAS-1

Sperm-specific proteins show generally elevated rates of evolution, consistent with strong selection on sperm functions because of sperm competition [63, 79]. The current data show that this is decisively the case for the sperm-specific variant HTAS-1. We show that the greater divergence of *C*. *elegans* HTAS-1 from core H2A is not because of differences in an evolutionary age, but very much despite it: HTAS-1 is most divergent variant protein despite being the most recent to diverge from core H2A, having since evolved at a rate many times higher than any other H2A paralog. This high rate of evolution strongly suggests that HTAS-1 may be adapted to play roles important for reproduction in some species. However, our data also have somewhat ambiguous implications for HTAS-1 function. On the one hand, though HTAS-1 has been maintained for long periods of time in many lineages it may have also been lost in multiple independent lineages, as found for HIS-35. While rapid evolution of HTAS-1 made it impossible to exclude the possibility that apparent HTAS-1 losses actually represent gene annotation failures, the fact that careful scrutiny revealed no gene annotation failures for HIS-35 weighs against the possibility of annotation failure, suggesting real loss of HTAS-1.

On the other hand, the much larger degree of protein sequence difference between HTAS-1 and H2A would seem to decrease the probability that HTAS-1 protein is functionally identical to H2A protein particularly given the extended C and N terminus of HTAS-1 which has previously been reported to play a vital role in DNA compaction, chromosome segregation, and fertility [63]. Moreover, the particular chromatin constraints of sperm production raise the possibility that HTAS-1 proteins could encode distinct functions relative to H2A proteins, for instance by leading to greater sperm DNA compaction; however, it is also possible that a distinct H2A paralog is simply necessary to ensure expression of H2A proteins well after germline mitosis in later stages of spermatogenesis that undergo transcription, DNA recombination and repair, and division. Thus, more study of the functional significance of HTAS-1 homologs in different species is clearly needed to distinguish between these possibilities.

### Concluding remarks

These results show exceptions to previously reported patterns, challenging sometimes implicit assumptions about non-core histones. First, whereas protein sequence differences between core and variant histone paralogs are often assumed to reflect differences in protein function, here we show that the variant protein HIS-35 is likely to have a redundant function with core H2A despite the sequence difference. Second, while all *C*. *elegans* H2A variants have a single intron, our observation of multi-intron variants and of recurrent intron loss, suggests that specific introns may not have crucial roles in the expression of histone variants. Instead, the role of introns in variant histones may simply lie in introns’ general roles in promoting expression. Third, the combination of conservation and loss of variant histones points to potentially lineage-specific, partially redundant, or easily replaced roles of some histone variants. Future studies should explore the generality of these patterns across other lineages of Eukaryotes. In addition to our specific findings for histone variant biology, these results highlight that introns can be useful in the reconstruction of the histories of complex gene families.

## Materials and methods

### Data source

Genomic sequences and gene feature format files of 108 nematode species were obtained from WormBase v11 and the *Caenorhabditis* database (caenorhabditis.org) (accessed May 1st 2019) [64, 65].

### Data mining and processing

All the known genes of 108 nematode species with characterized exon-intron structures were fetched from their genomes using their respective gff annotation files. We then annotated the positions of the introns in the header of their respective genes and translated the *Caenorhabditis* gene sequences.

To identify the homologs of H2A and their variants, BLASTP, version 2.9.0+, was performed using standard parameters while treating the translated gene sequences (of 108 nematode species) as the database and H2A and variant (H2A.Z^HTZ-1^, HTAS-1, HIS-35) protein sequences as the query [80]. Using a maximum e-value of 1e-10, 8003 hits were retrieved which were the homologs of H2A and H2A-variant genes. We then removed dubious genes encoding proteins more than 200 amino acids long, because histone proteins are generally shorter. We collapsed the genes whose introns align at the same position and that have an identical protein sequence. After filtering, we were left with 408 distinct protein entries. For generating a nucleotide dataset, the same filtering was performed at the protein level except unique nucleotide sequences were kept instead of being collapsed by their identical amino acid sequence identities, resulting in 593 DNA sequences.

Previous studies have shown the intron position conservation among widely diverged eukaryotic species [52–54, 56, 68, 69, 77]. Therefore, to assess the intron position conservation among the putative H2A variant genes, we performed a Multiple Sequence Alignment (MSA) using the default parameters of CLUSTALW 2.1 [81]. We mapped the intron positions of each gene onto the corresponding protein CLUSTALW alignment, allowing us to identify as potential H2A.Z^HTZ-1^, HTAS-1, HIS-35 orthologs those genes with intron positions matching *C*. *elegans* intron positions.

### Phylogenetic analyses

To reconstruct the relationships of all homologs of H2A and its variants, we generated both protein and nucleotide-based trees. The 408 protein homologs were aligned using default parameters of MAFFT v7.307 [82]. The S2 Fig in S2 File midpoint rooted Maximum Likelihood protein tree was generated using IQ-TREE v1.6.10, which does an automatic selection of the model by doing a model fit test and likelihood scoring and here selected the VT+R8 model, and 10,000 bootstrap replicates were used to produce branch supports [83].

The 593 nucleotide sequences for the proteins included in S2 Fig in S2 File were aligned by back-translating the MAFFT protein alignments into their original codons, maintaining the alignment based on amino acid sequence. IQ-TREE was used to generate a Maximum Likelihood phylogenetic tree with a codon-based model; automatic model selection was again used and the model KOSI07+FU+R10 was automatically selected. Branch supports were generated by 10,000 bootstrap replicates in IQ-TREE and the tree is displayed midpoint rooted (S3 Fig in S2 File).

Both trees did not yield a clear phylogenetic signal for HIS-35 or H2A.Z^HTZ-1^, with homologs exhibiting the *C*. *elegans* HIS-35 or H2A.Z^HTZ-1^ intron positions are scattered over the tree (S2 and S3 Figs in S2 File; IP 234 in yellow and IP 236 in purple, respectively). However, when we took a closer look at the HIS-35 characteristic intron-containing genes, we noted that genes containing an intron at the *C*. *elegans* HIS-35 intron position (S2 and S3 Figs in S2 File, IP 234, yellow) were restricted to most species of *Caenorhabditis* and its sister genus *Diploscapter*. A clear clade of species was seen which had HTAS-1 characteristic intron position (S2 and S3 Figs in S2 File, IP 152, green). For consistency in Figs 2–4, we show the portion of the phylogenetic tree containing *Caenorhabditis* species and *Disploscapter*.

To attempt to gain further clarity on the relationships between core H2A and HIS-35, we took several approaches. First, a phylogenetic tree based on core H2A and HIS-35 protein orthologs for *Caenorhabditis* and *Diploscaptor* species was generated. Alignments were performed in MAFFT and midpoint rooted Maximum Likelihood trees were generated in IQ-TREE with the automatically selected VT+R5 model and 1000 bootstrap replicates.

To investigate cases of concerted evolution (discussed above) between H2A and HIS-35, we reconstructed phylogenetic trees for the first and second exons separately for all *Caenorhabditis* H2A and HIS-35 sequences. Alignments of individual exonic nucleotide sequences were performed with CLUSTALW and IQ-TREE with automatic model selection and 1000 bootstrap replicates. For exon 1, the TIM2e+I+G4 model was selected (S5 Fig in S2 File) and for exon 2, the TIM3e+G4 model was selected (S6 Fig in S2 File).

All phylogenetic trees were visualized in Figtree v1.4.4 [84].

### Confirmation of H2A variant losses

We found a loss of H2A.Z^HTZ-1^, HTAS-1, HIS-35 characteristic introns in a few lineages (marked by a minus sign in Figs 2–4). To know whether these represent real losses or reflect errors in gene annotation, TBLASTN searches were performed across the genome of these species. This manual curation led to the characteristic intron splice sites of the variants being identified by eye in a few species due to alignment gaps at the exact intron position, indicating that these species truly contain the variant and that failure to initially identify the variant is due to a failure of the annotation to include these genes. To further investigate legitimate gene loss versus technical issues, BUSCO v5.5.0 [70] was used to assess *Caenorhabditis* species and the outgroup *Diploscaptor coronatus* genome assembly qualities and completeness using the nematode odb10 database for reference (S1 Fig in S2 File). To test the possibility that inferred gene losses of HIS-35 and HTAS-1 in various *Caenorhabditis* species are the result of poor genome assemblies rather than real loss, we performed one-tailed t-tests on BUSCO completeness and missing gene estimates. We included a materials and methods flowchart for the general analysis approach as S7 Fig in S2 File.

## Supporting information

S1 File (DOCX)

S2 File (PDF)
