# Supplementary material for: Nematode histone H2A variant evolution reveals diverse histories of retention and loss and evidence for conserved core-like variant histone genes
Source: PLoS One. 2024 May 30;19(5):e0300190. doi: 10.1371/journal.pone.0300190 (PMC11139335; doi:10.1371/journal.pone.0300190)
Supplement: S2 File — (PDF) [file pone.0300190.s002.pdf]

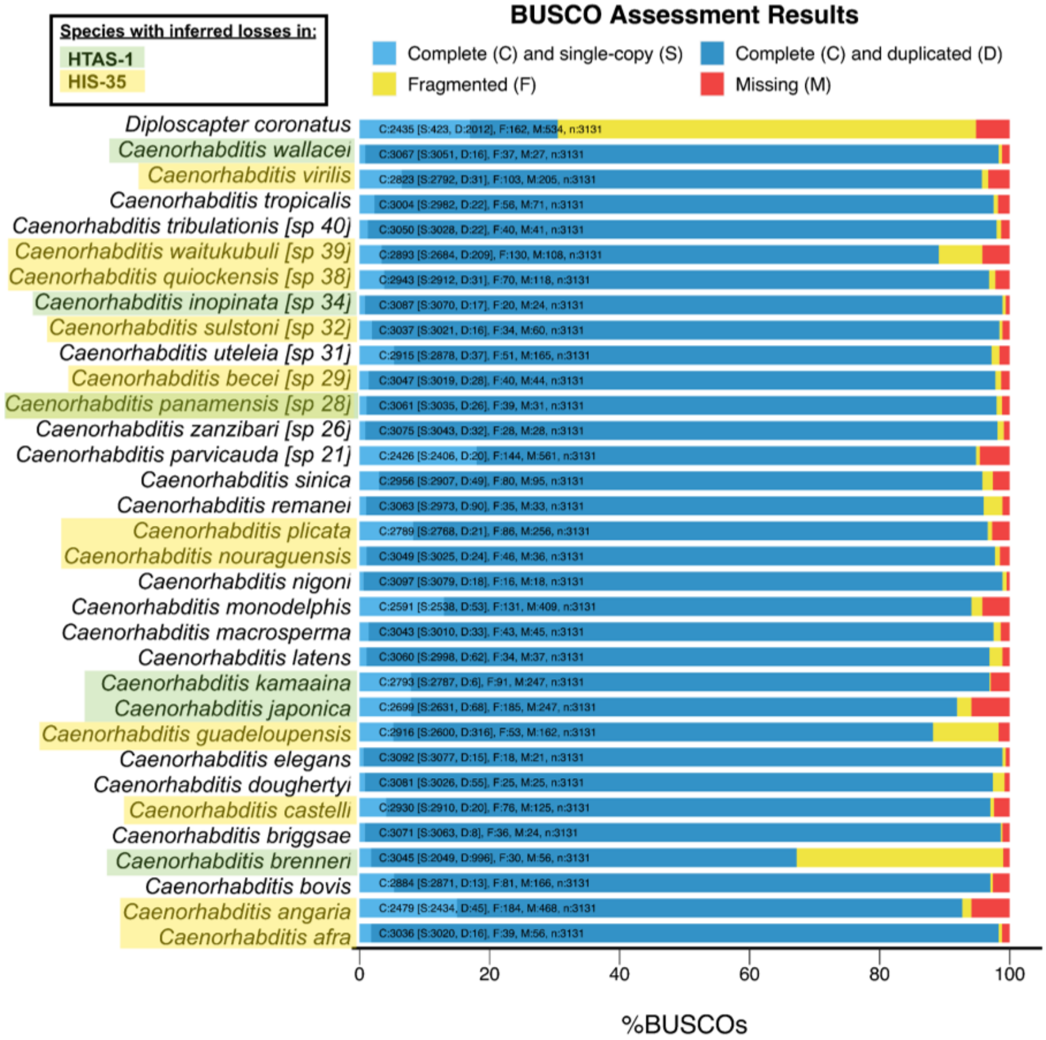

Figure S1

BUSCO report for *Caenorhabditis* species and the *Diploscaptor coronatus* outgroup genome assemblies. Species are annotated by whether we infer HTAS-1 (green highlight) or HIS-35 (yellow highlight) gene losses in these species, corresponding with those shown in main text Figures 2 and 3-left. *Caenorhabditis panamensis* [sp. 28] is shaded for both as we infer loss of HTAS-1 and HIS-35. The % BUSCO represents the percentage of genes in the nematode odb10 database present in each genome assembly. Counts for each BUSCO gene category are shown inside the bars. By t-test we do not find that *Caenorhabditis* species with inferred losses have poorer quality or more incomplete genomes compared to *Caenorhabditis* species and *Diploscaptor coronatus* with intact gene copies [HTAS-1: Completeness ($p = 0.3243$), Missing ($p = 0.3605$); HIS-35: Completeness ($p = 0.4203$), Missing ($p = 0.4781$)]. Genome assemblies used here are from WormBase v11 and the Caenorhabditis database (caenorhabditis.org) (accessed May 1st 2019) (67, 68).

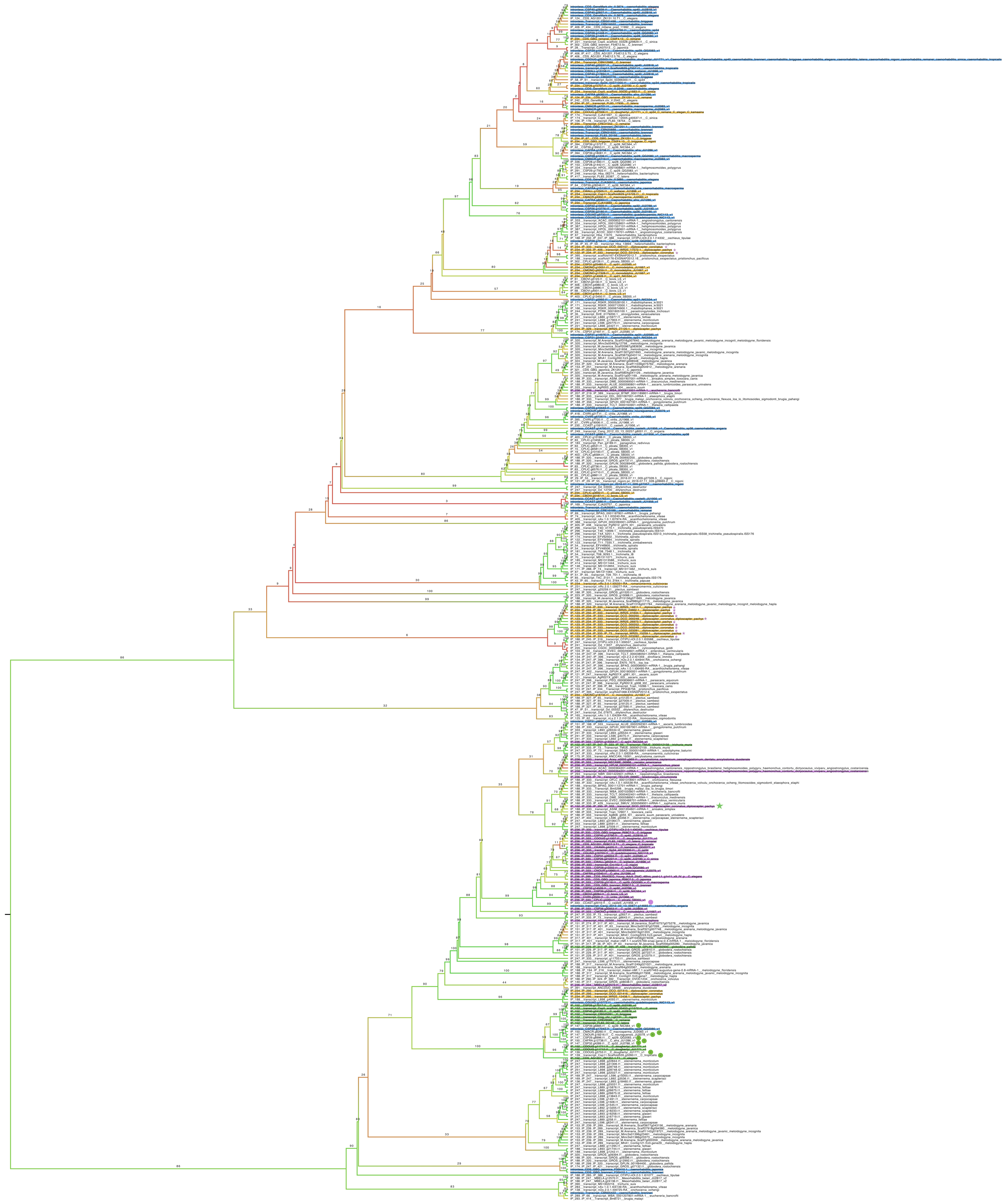

Figure S2

Cladogram reconstructed from protein sequences encoded by H2A and H2A-related variant genes across 108 available nematode genomes. The tree is made up of 408 unique protein entries, with genes encoding identical protein sequences and with identical intron positions collapsed to a single entry (in which cases all collapsed species are indicated). Alignments performed with MAFFT v7.307 were used for Maximum Likelihood analysis in IQ-TREE v1.6.10 using the program-selected VT+R8 model and with 10,000 bootstrap replicates. The tree is rooted at its midpoint and branches are colored by bootstrap support, also shown at nodes, with a gradient from red (poorer support) to green (higher support) with the inflection point toward green at BS = 60.

On the tree, “IP” followed by a number stands for the position at which the intron was aligned during the multiple sequence alignment. Intronless sequences (most of which are likely canonical H2As) are indicated as “intronless” and highlighted in blue. The intron position of *C. elegans* HTAS-1 is 152 (IP 152), highlighted in green. The intron position of *C. elegans* HIS-35 is 234 (IP 234), highlighted in yellow, and the intron position of the *C. elegans* HTZ-1 is 236 (IP 236) and highlighted in purple.

Purple stars indicate sequences with *C. elegans* intron positions indicating non-HTZ-1 sequences (indicated by shading) but that also share the second HTZ-1 intron position, IP 333. The green star indicates a sequence with both the *C. elegans* HTZ-1 position 236 and the *C. elegans* HTAS-1 position 152 that is more likely HTZ-1 because of its phylogenetic context. The purple circle indicates a likely HTZ-1 sequence, by its phylogenetic position and the presence of the second intron position 333 (*C. castelli* has lost the first intron position, see main text Figure 4). Green circles indicate likely HTAS-1 sequences by their phylogenetic positions and manual sequence verifications via TBLASTN (See main text).

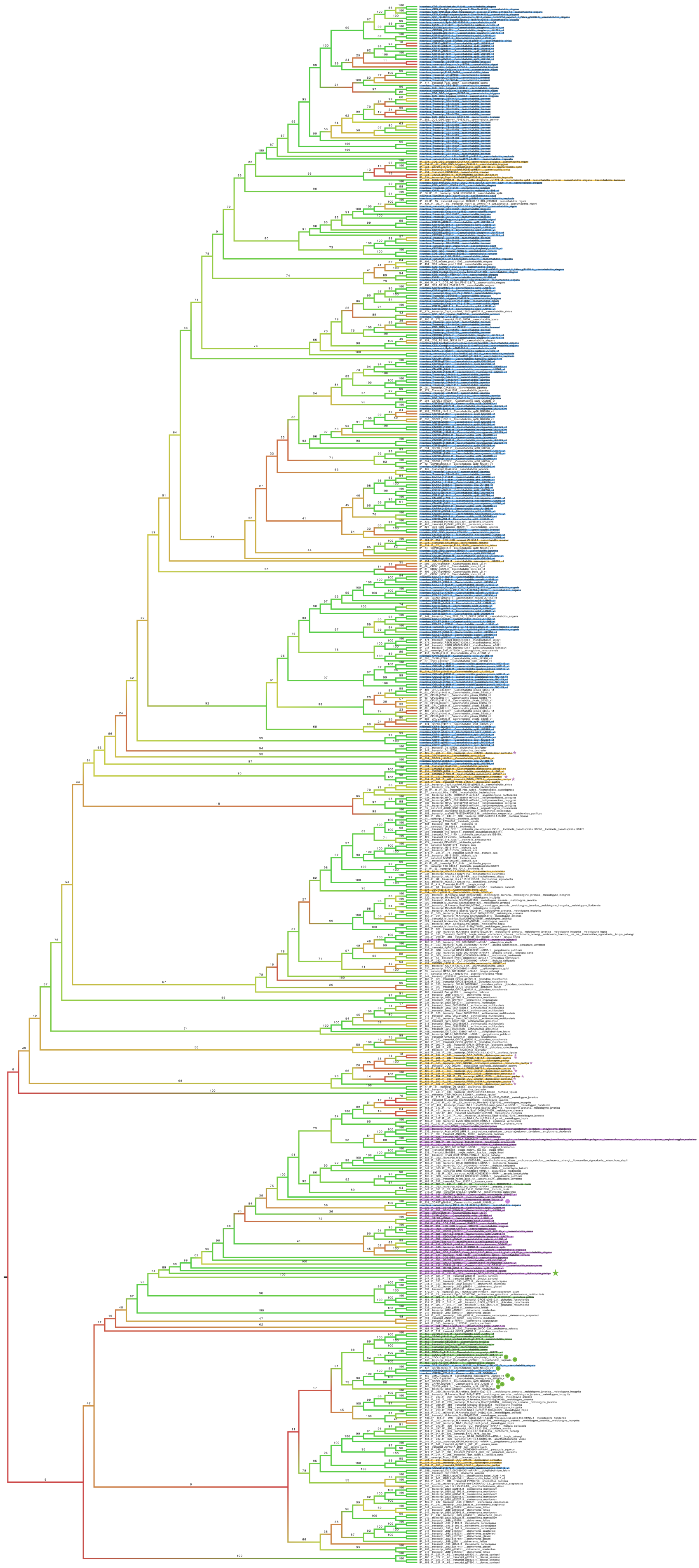

Figure S3
Cladogram reconstructed from nucleotide sequences encoded by H2A and H2A-related variant genes across 108 available nematode genomes. The tree is made up of 593 unique nucleotide entries and is rooted by its midpoint. Alignments performed with MAFFT v7.307 were used for Maximum Likelihood analysis in IQ-TREE v1.6.10 where a codon-based model was specified (KOSI07+FU+R10 was automatically selected per BIC) and 10,000 bootstrap replicates were generated. Branches are colored by bootstrap support, also shown at nodes, with a gradient from red (poorer support) to green (higher support) with the inflection point toward green at BS = 60.

On the tree, “IP” followed by a number stands for the position at which the intron was aligned during the multiple sequence alignment. Intronless sequences (most of which are likely canonical H2As) are indicated as “intronless” and highlighted in blue. The intron position of *C. elegans* HTAS-1 is 152 (IP 152), highlighted in green. The intron position of *C. elegans* HIS-35 is 234 (IP 234), highlighted in yellow, and the intron position of the *C. elegans* HTZ-1 is 236 (IP 236) and highlighted in purple.

Purple stars indicate sequences with *C. elegans* intron positions indicating non-HTZ-1 sequences (indicated by shading) but that also share the second HTZ-1 intron position, IP 333. The green star indicates a sequence with both the *C. elegans* HTZ-1 position 236 and the *C. elegans* HTAS-1 position 152 that is more likely HTZ-1 because of its phylogenetic context. The purple circle indicates a likely HTZ-1 sequence, by its phylogenetic position and the presence of the second intron position 333 (*C. castelli* has lost the first intron position, see main text Figure 4). Green circles indicate likely HTAS-1 sequences by their phylogenetic positions and manual sequence verifications via TBLASTN (See main text).

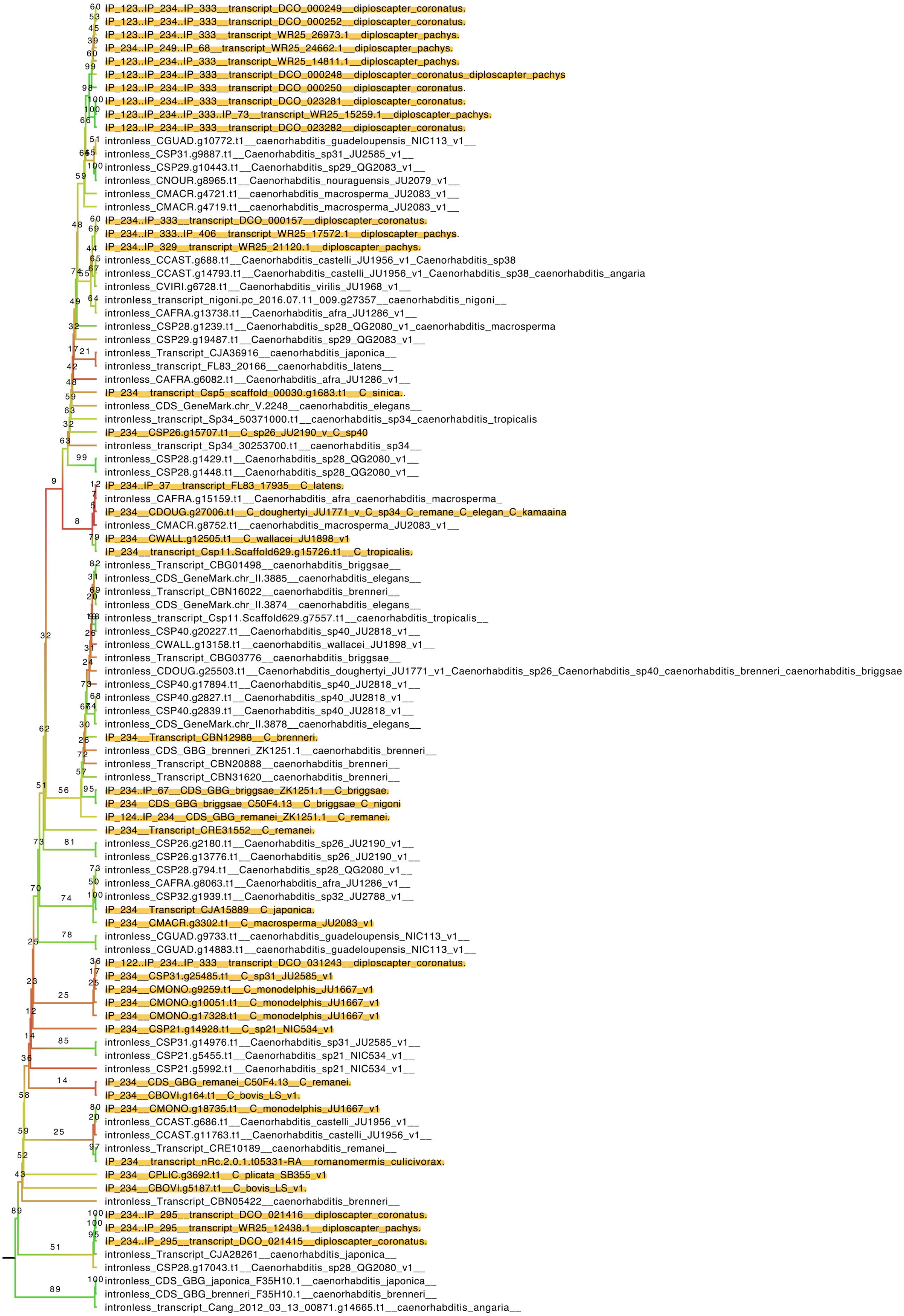
IP_123..IP_234..IP_333__transcript_DCO_000249__diploscapter_coronatus.
IP_123..IP_234..IP_333__transcript_DCO_000252__diploscapter_coronatus.
IP_123..IP_234..IP_333__transcript_WR25_26973.1__diploscapter_pachys.
IP_234..IP_249..IP_68__transcript_WR25_24662.1__diploscapter_pachys.
IP_123..IP_234..IP_333__transcript_WR25_14811.1__diploscapter_pachys.
IP_123..IP_234..IP_333__transcript_DCO_000248__diploscapter_coronatus_diploscapter_pachys
IP_123..IP_234..IP_333__transcript_DCO_000250__diploscapter_coronatus.
IP_123..IP_234..IP_333__transcript_DCO_023281__diploscapter_coronatus.
IP_123..IP_234..IP_333..IP_73__transcript_WR25_15259.1__diploscapter_pachys.
IP_123..IP_234..IP_333__transcript_DCO_023282__diploscapter_coronatus.
intronless_CGUAD.g10772.t1__caenorhabditis_guadeloupensis_NIC113_v1__
intronless_CSP31.g9887.t1__Caenorhabditis_sp31_JU2585_v1__
intronless_CSP29.g10443.t1__Caenorhabditis_sp29_QG2083_v1__
intronless_CNOUR.g8965.t1__Caenorhabditis_nouraguensis_JU2079_v1__
intronless_CMACR.g4721.t1__caenorhabditis_macrosperma_JU2083_v1__
intronless_CMACR.g4719.t1__caenorhabditis_macrosperma_JU2083_v1__
IP_234..IP_333__transcript_DCO_000157__diploscapter_coronatus.
IP_234..IP_333..IP_406__transcript_WR25_17572.1__diploscapter_pachys.
IP_234..IP_329__transcript_WR25_21120.1__diploscapter_pachys.
intronless_CCAST.g688.t1__Caenorhabditis_castelli_JU1956_v1_Caenorhabditis_sp38
intronless_CCAST.g14793.t1__Caenorhabditis_castelli_JU1956_v1_Caenorhabditis_sp38_caenorhabditis_angaria
intronless_CVIRI.g6728.t1__Caenorhabditis_virilis_JU1968_v1__
intronless_transcript_nigoni.pc_2016.07.11_009.g27357__caenorhabditis_nigoni__
intronless_CAFRA.g13738.t1__Caenorhabditis_afra_JU1286_v1__
intronless_CSP28.g1239.t1__Caenorhabditis_sp28_QG2080_v1_caenorhabditis_macrosperma
intronless_CSP29.g19487.t1__Caenorhabditis_sp29_QG2083_v1__
intronless_Transcript_CJA36916__caenorhabditis_japonica__
intronless_transcript_FL83_20166__caenorhabditis_latens__
intronless_CAFRA.g6082.t1__Caenorhabditis_afra_JU1286_v1__
IP_234__transcript_Csp5_scaffold_00030.g1683.t1__C_sinica..
intronless_CDS_GeneMark.chr_V.2248__caenorhabditis_elegans__
intronless_transcript_Sp34_50371000.t1__caenorhabditis_sp34_caenorhabditis_tropicalis
IP_234__CSP26.g15707.t1__C_sp26_JU2190_v_C_sp40
intronless_transcript_Sp34_30253700.t1__caenorhabditis_sp34__
intronless_CSP28.g1429.t1__Caenorhabditis_sp28_QG2080_v1__
intronless_CSP28.g1448.t1__Caenorhabditis_sp28_QG2080_v1__
IP_234..IP_37__transcript_FL83_17935__C_latens.
intronless_CAFRA.g15159.t1__Caenorhabditis_afra_caenorhabditis_macrosperma_
IP_234__CDOUG.g27006.t1__C_doughertyi_JU1771_v_C_sp34_C_remane_C_elegan_C_kamaaina
intronless_CMACR.g8752.t1__caenorhabditis_macrosperma_JU2083_v1__
IP_234__CWALL.g12505.t1__C_wallacei_JU1898_v1
IP_234__transcript_Csp11.Scaffold629.g15726.t1__C_tropicalis.
intronless_Transcript_CBG01498__caenorhabditis_briggsae__
intronless_CDS_GeneMark.chr_II.3885__caenorhabditis_elegans__
intronless_Transcript_CBN16022__caenorhabditis_brenneri__
intronless_CDS_GeneMark.chr_II.3874__caenorhabditis_elegans__
intronless_transcript_Csp11.Scaffold629.g7557.t1__caenorhabditis_tropicalis__
intronless_CSP40.g20227.t1__Caenorhabditis_sp40_JU2818_v1__
intronless_CWALL.g13158.t1__caenorhabditis_wallacei_JU1898_v1__
intronless_Transcript_CBG03776__caenorhabditis_briggsae__
intronless_CDOUG.g25503.t1__Caenorhabditis_doughertyi_JU1771_v1_Caenorhabditis_sp26_Caenorhabditis_sp40_caenorhabditis_brenneri_caenorhabditis_briggsae
intronless_CSP40.g17894.t1__Caenorhabditis_sp40_JU2818_v1__
intronless_CSP40.g2827.t1__Caenorhabditis_sp40_JU2818_v1__
intronless_CSP40.g2839.t1__Caenorhabditis_sp40_JU2818_v1__
intronless_CDS_GeneMark.chr_II.3878__caenorhabditis_elegans__
IP_234__Transcript_CBN12988__C_brenneri.
intronless_CDS_GBG_brenneri_ZK1251.1__caenorhabditis_brenneri__
intronless_Transcript_CBN20888__caenorhabditis_brenneri__
intronless_Transcript_CBN31620__caenorhabditis_brenneri__
IP_234..IP_67__CDS_GBG_briggsae_ZK1251.1__C_briggsae.
IP_234__CDS_GBG_briggsae_C50F4.13__C_briggsae_C_nigoni
IP_124..IP_234__CDS_GBG_remanei_ZK1251.1__C_remanei.
IP_234__Transcript_CRE31552__C_remanei.
intronless_CSP26.g2180.t1__Caenorhabditis_sp26_JU2190_v1__
intronless_CSP26.g13776.t1__Caenorhabditis_sp26_JU2190_v1__
intronless_CSP28.g794.t1__Caenorhabditis_sp28_QG2080_v1__
intronless_CAFRA.g8063.t1__Caenorhabditis_afra_JU1286_v1__
intronless_CSP32.g1939.t1__Caenorhabditis_sp32_JU2788_v1__
IP_234__Transcript_CJA15889__C_japonica.
IP_234__CMACR.g3302.t1__C_macrosperma_JU2083_v1
intronless_CGUAD.g9733.t1__caenorhabditis_guadeloupensis_NIC113_v1__
intronless_CGUAD.g14883.t1__caenorhabditis_guadeloupensis_NIC113_v1__
IP_122..IP_234..IP_333__transcript_DCO_031243__diploscapter_coronatus.
IP_234__CSP31.g25485.t1__C_sp31_JU2585_v1
IP_234__CMONO.g9259.t1__C_monodelphis_JU1667_v1
IP_234__CMONO.g10051.t1__C_monodelphis_JU1667_v1
IP_234__CMONO.g17328.t1__C_monodelphis_JU1667_v1
IP_234__CSP21.g14928.t1__C_sp21_NIC534_v1
intronless_CSP31.g14976.t1__Caenorhabditis_sp31_JU2585_v1__
intronless_CSP21.g5455.t1__Caenorhabditis_sp21_NIC534_v1__
intronless_CSP21.g5992.t1__Caenorhabditis_sp21_NIC534_v1__
IP_234__CDS_GBG_remanei_C50F4.13__C_remanei.
IP_234__CBOVI.g164.t1__C_bovis_LS_v1.
IP_234__CMONO.g18735.t1__C_monodelphis_JU1667_v1
intronless_CCAST.g686.t1__Caenorhabditis_castelli_JU1956_v1__
intronless_CCAST.g11763.t1__Caenorhabditis_castelli_JU1956_v1__
intronless_Transcript_CRE10189__caenorhabditis_remanei__
IP_234__transcript_nRc.2.0.1.t05331-RA__romanomermis_culicivorax.
IP_234__CPLIC.g3692.t1__C_plicata_SB355_v1
IP_234__CBOVI.g5187.t1__C_bovis_LS_v1.
intronless_Transcript_CBN05422__caenorhabditis_brenneri__
IP_234..IP_295__transcript_DCO_021416__diploscapter_coronatus.
IP_234..IP_295__transcript_WR25_12438.1__diploscapter_pachys.
IP_234..IP_295__transcript_DCO_021415__diploscapter_coronatus.
intronless_Transcript_CJA28261__caenorhabditis_japonica__
intronless_CSP28.g17043.t1__Caenorhabditis_sp28_QG2080_v1__
intronless_CDS_GBG_japonica_F35H10.1__caenorhabditis_japonica__
intronless_CDS_GBG_brenneri_F35H10.1__caenorhabditis_brenneri__
intronless_transcript_Cang_2012_03_13_00871.g14665.t1__caenorhabditis_angaria__

Figure S4
Maximum Likelihood midpoint rooted cladogram of core H2A orthologs and HIS-35 orthologs identified based on intron positions for *Caenorhabditis and Diploscaptor* species, generated with the automatically chosen VT+R5 model in IQ-TREE v1.6.10 with 1,000 bootstrap replicates based on an alignment with MAFFT v7.307. Branches are colored by bootstrap support, also shown at nodes, with a gradient from red (poorer support) to green (higher support) with the inflection point toward green at BS = 60. HIS-35 sequences are shaded in yellow. Here, we expect distinct clades of H2A and HIS-35 sequences, yet we recover no such separate clades.

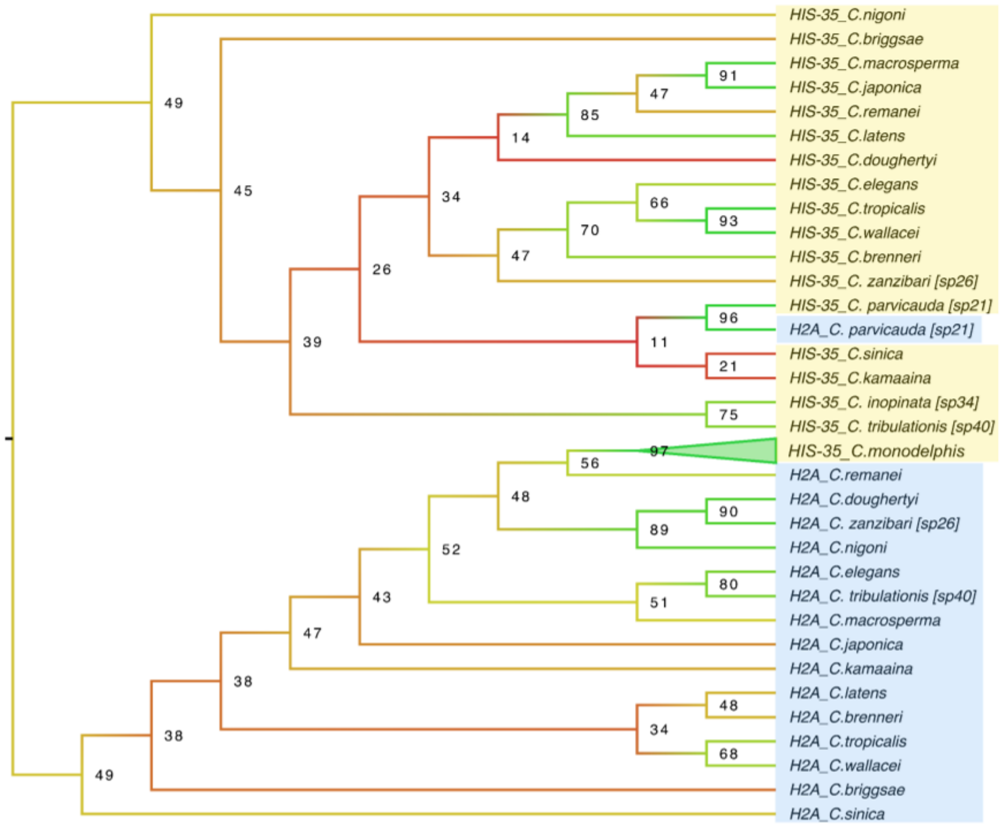

Figure S5

Cladogram reconstructed from nucleotide sequences of canonical *Caenorhabditis* H2A gene sequences (blue shaded) and first exons of candidate HIS-35 gene sequences (yellow shaded). Alignments performed with CLUSTALW 2.1 were used for Maximum Likelihood analysis in IQ-TREE v1.6.10 using the program-selected TIM2e+I+G4 model and with 1,000 bootstrap replicates. The tree was rooted on the majority-H2A clade. Branches are colored by bootstrap support, also shown at nodes, with a gradient from red (poorer support) to green (higher support) with the inflection point toward green at BS = 60. We see clear clusters of H2A nucleotide sequences and HIS-35 with one exception, *Caenorhabditis parvicauda [sp 21]*, discussed in main text.

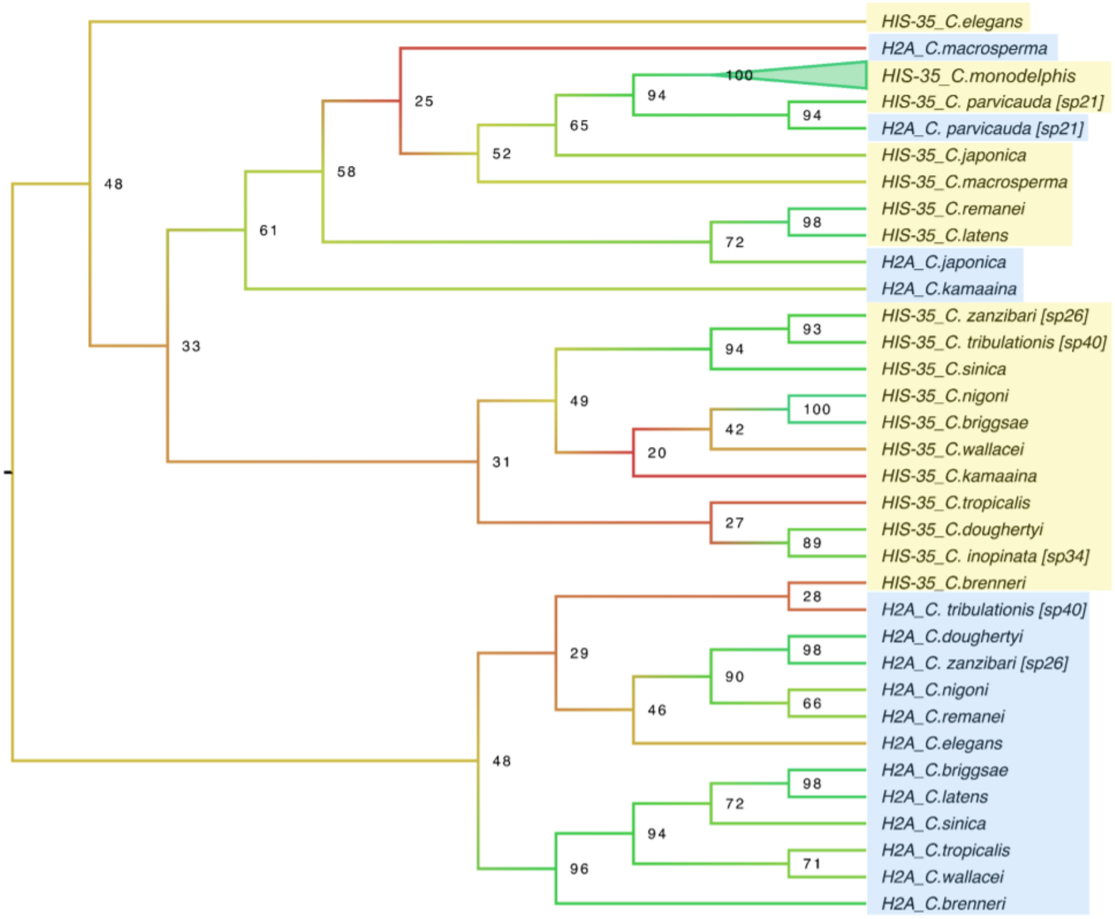

Figure S6

Cladogram reconstructed from nucleotide sequences of canonical *Caenorhabditis* H2A gene sequences (blue shaded) and second exons of candidate HIS-35 gene sequences (yellow shaded). Alignments performed with CLUSTALW 2.1 were used for Maximum Likelihood analysis in IQ-TREE v1.6.10 using the program-selected TIM3e+G4 model and with 1,000 bootstrap replicates. The tree was rooted on the majority-H2A clade. Branches are colored by bootstrap support, also shown at nodes, with a gradient from red (poorer support) to green (higher support) with the inflection point toward green at BS = 60. The second exon used here less stably reconstructs clades than the first exon in Figure S5, though a majority H2A group is recovered.

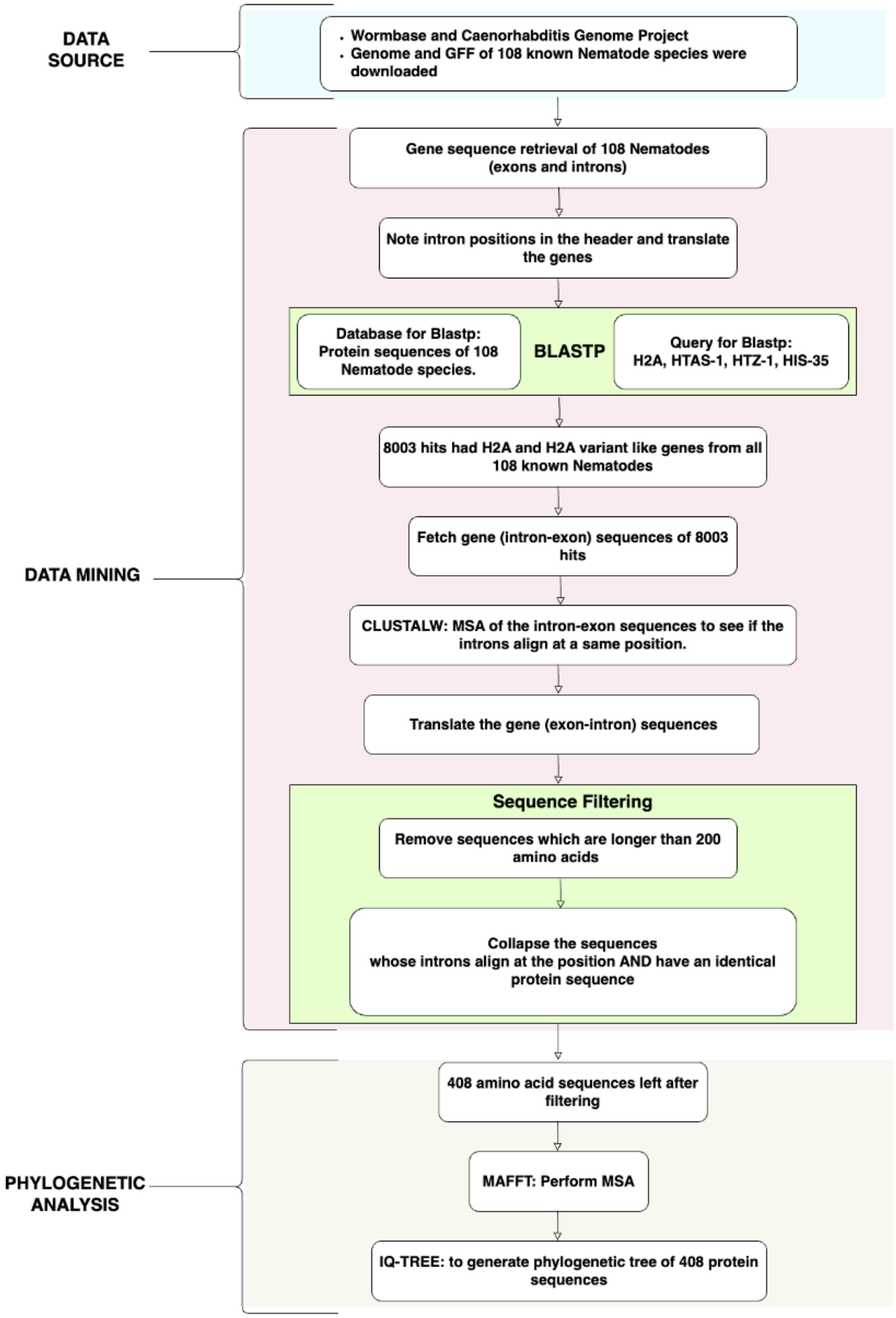

Figure S7

Material and methods flowchart for main analysis.
